# Optical genome mapping in an atypical Pelizaeus-Merzbacher prenatal challenge

**DOI:** 10.3389/fgene.2023.1173426

**Published:** 2023-07-25

**Authors:** Mihael Rogac, Anja Kovanda, Luca Lovrečić, Borut Peterlin

**Affiliations:** ^1^ Clinical Institute of Genomic Medicine, University Medical Center Ljubljana, Ljubljana, Slovenia; ^2^ Faculty of Medicine, University of Ljubljana, Ljubljana, Slovenia

**Keywords:** Pelizaeus-Merzbacher disease, *PLP1*, proteolipid protein gene, optical genome mapping, microarray, inverted duplication, Xq22.2

## Abstract

Pathogenic genetic variants represent a challenge in prenatal counseling, especially when clinical presentation in familial carriers is atypical. We describe a prenatal case involving a microarray-detected duplication of *PLP1* which causes X-linked Pelizaeus-Merzbacher disease, a progressive hypomyelinating leukodystrophy. Because of atypical clinical presentation in an older male child, the duplication was examined using a novel technology, optical genome mapping, and was found to be an inverted duplication, which has not been previously described. Simultaneously, segregation analysis identified another healthy adult male carrier of this unique structural rearrangement. The novel *PLP1* structural variant was reclassified, and a healthy boy was delivered. In conclusion, we suggest that examining structural variants with novel methods is warranted especially in cases with atypical clinical presentation and may in these cases lead to improved prenatal and postnatal genetic counseling.

## Introduction

Pelizaeus-Merzbacher disease (PMD, OMIM:312080) is a severe X-linked recessive hypomyelinating leukodystrophy, with initial clinical presentation of nystagmus and developmental delay, progressing to spastic quadriplegia and ataxia (Sistermans et al., 1998). PMD is caused by disease-causing variants in the proteolipid protein gene (*PLP1*, OMIM:300401), resulting in the improper formation of myelin in the central nervous system (Wolf et al., 1993).

Gene duplications of *PLP1* represent a common cause of PMD and account for 50%–75% of all clinically manifest disease-causing variants and yield the classic PMD phenotype (Garbern et al., 1999; Osório and Goldman, 2018). Both triplosensitivity and haploinsufficiency of the *PLP1* gene have been well-established (Raskind et al., 1991; Woodward et al., 1998; Mimault et al., 1999; Cailloux et al., 2000; Hobson et al., 2000; Inoue et al., 2002; Wolf et al., 2005; Combes et al., 2006; Regis et al., 2009; Grossi et al., 2011). Molecular genetic testing of *PLP1* disorders is usually performed by gene-targeted deletion/duplication analysis using multiplex ligation-dependent probe amplification (MLPA), microarray, quantitative PCR, long-range PCR, fluorescent *in situ* hybridization, and combinations thereof (Wolf et al., 1993), however these methods are not designed to detect both the orientation and additional genomic context of the affected region. While male PMD patients typically do not reproduce, female carriers of pathogenic variants carry a 50% chance of transmitting the variant in each pregnancy, and prenatal testing for a pregnancy at increased risk is available if the pathogenic variant is known (Wolf et al., 1993).

We now present our prenatal *PLP1* duplication case, where the older male child of the carrier mother had an atypical neuro-developmental clinical presentation inconsistent with the microarray-detected PMD pathogenic duplication, which prompted the analyses of this structural variant using a novel laboratory technology, optical genome mapping (OGM) (Dremsek et al., 2021; Mantere et al., 2021). Based on the findings of OGM which showed a complex variant involving an inverted duplication of *PLP1*, and extended segregation analysis, this novel structural variant was reclassified, and a healthy child was delivered.

## Clinical presentation and laboratory findings

A woman planning a pregnancy was referred for antenatal genetic consultation to the Clinical Institute of Genomic Medicine (CIGM), University Medical Centre Ljubljana (UMCL), Slovenia, because of clinical problems in her older child. Because of the pandemic, she received a teleconsultation. Her first child, a 7-year-old boy reportedly had an unspecific short attention span, mild central, and axial hypotonia, elements of autistic features, and joint hypermobility. The perinatal history of the boy was unremarkable as reported by the parents via teleconsultation and the boy was referred for genetic testing using microarray.

**FIGURE 1:**
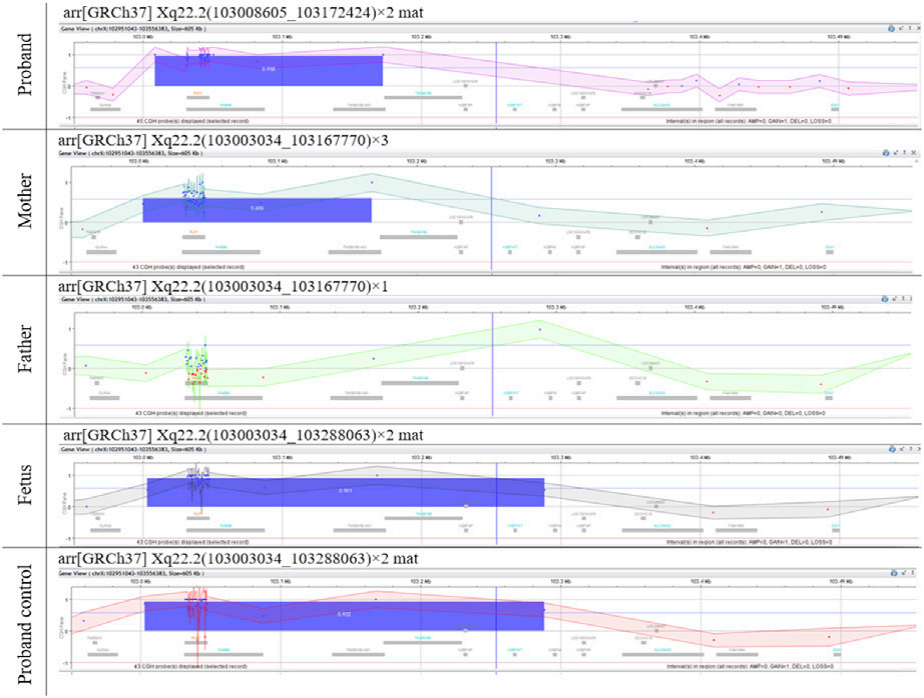
Microarray results. Microarray analyses of the genomic gain of Xq22.2 region containing *PLP1* in the family. The differences in the reported sizes of the duplicated region are due to the different array design used and were confirmed by control microarray analyses (Supplementary Figure S1).

The mother was already in the first trimester of pregnancy by the time microarray was performed on her son. The microarray analysis revealed a duplication at arr[GRCh37] Xq22.2(103008605_103172424)×2, approximately 163.8 kb in size (Figure 1). The duplication included the disease-associated *PLP1* gene (OMIM:300401), the duplication of which was previously reported as pathogenic and associated with PMD, and the *RAB9* (OMIM:300285) gene, that has not yet been associated with any disease. The reported clinical presentation in the proband was atypical of PMD, therefore, we performed segregation by microarray on the parents, which revealed the mother to be a carrier of the *PLP1* duplication (164.7 kb(arr[GRCh37] Xq22.2(103003034_103167770)×3]. Subsequently, amniocentesis was performed, and the presence of the duplication was also confirmed in a male fetus (Figure 1). Due to the difference between postnatal and prenatal array design, the reported size of the duplication in the fetus was approx. 285.0 kb (arr[GRCh37] Xq22.2(103003034_103288063)×2 or arr[GRCh38] Xq22.2(103748106_104033500)×2], and additionally includes gene *TMSB15B* (OMIM:301011), that has not yet been associated with any disease. The equal size of the variant in the proband was confirmed by a control array of the proband on the prenatal chip design (Figure 1).

However, the atypical clinical presentation of the proband made us further question the validity of the interpretation of this familial variant as pathogenic.

This presented a unique prenatal counseling challenge, as the non-specific and non-severe clinical manifestation in the older child did not indicate the need for a pregnancy intervention despite the detection of a pathogenic variant for a severe, progressive, early-onset disorder.

Indeed, the review of the proband’s medical history, the clinical re-examination, and the repeated MRI scan confirmed an atypical clinical presentation, inconsistent with PMD. Although behavioral issues were reported in kindergarten, and initial evaluation for autism spectrum disorder confirmed elements of autistic behavior, at the time of examination the proband displayed a short attention span and joint hypermobility, but apart from this, successfully attended regular primary school. Furthermore, the MRI displayed sequelae of T2 and FLAIR hyperintensities in the right hemisphere, resulting from a head injury at age 8 months that required surgical intervention, but showed no signs of hypomyelination that would be consistent with PMD.

To investigate the nature of the identified *PLP1* duplication further, we tested the proband by using a novel laboratory technology, optical genome mapping (OGM). OGM has been available for routine genetic testing in Slovenia since 2021 and enables the detection of structural genomic variants, such as inversions, duplications, insertions, deletions, translocations, and complex rearrangements in DNA size ranges previously undetectable by other technologies.

Since the carrier mother was from a large family with six neurologically normal brothers, we were able, in parallel, to perform segregation analysis to find possible additional unaffected male carriers of the familial variant.

Optical genome mapping analysis of the proband revealed the genomic gain in the Xq22.2, to be approximately 432.7 kb in size. The variant is a previously undescribed complex structural variant of *PLP1*—an inverted duplication of region [GRCh38] Xq22.2(103734669_103958435)×2 approx. 223.8 kb in size, followed by tandem duplication of region [GRCh38] Xq22.2(103871512_104080423)×2 approx. 208.9 kb in size. This inverted duplication, followed by the tandem duplication, is inserted at approximate position [GRCh38] Xq22.2(104080423_104085533).

**FIGURE 2:**
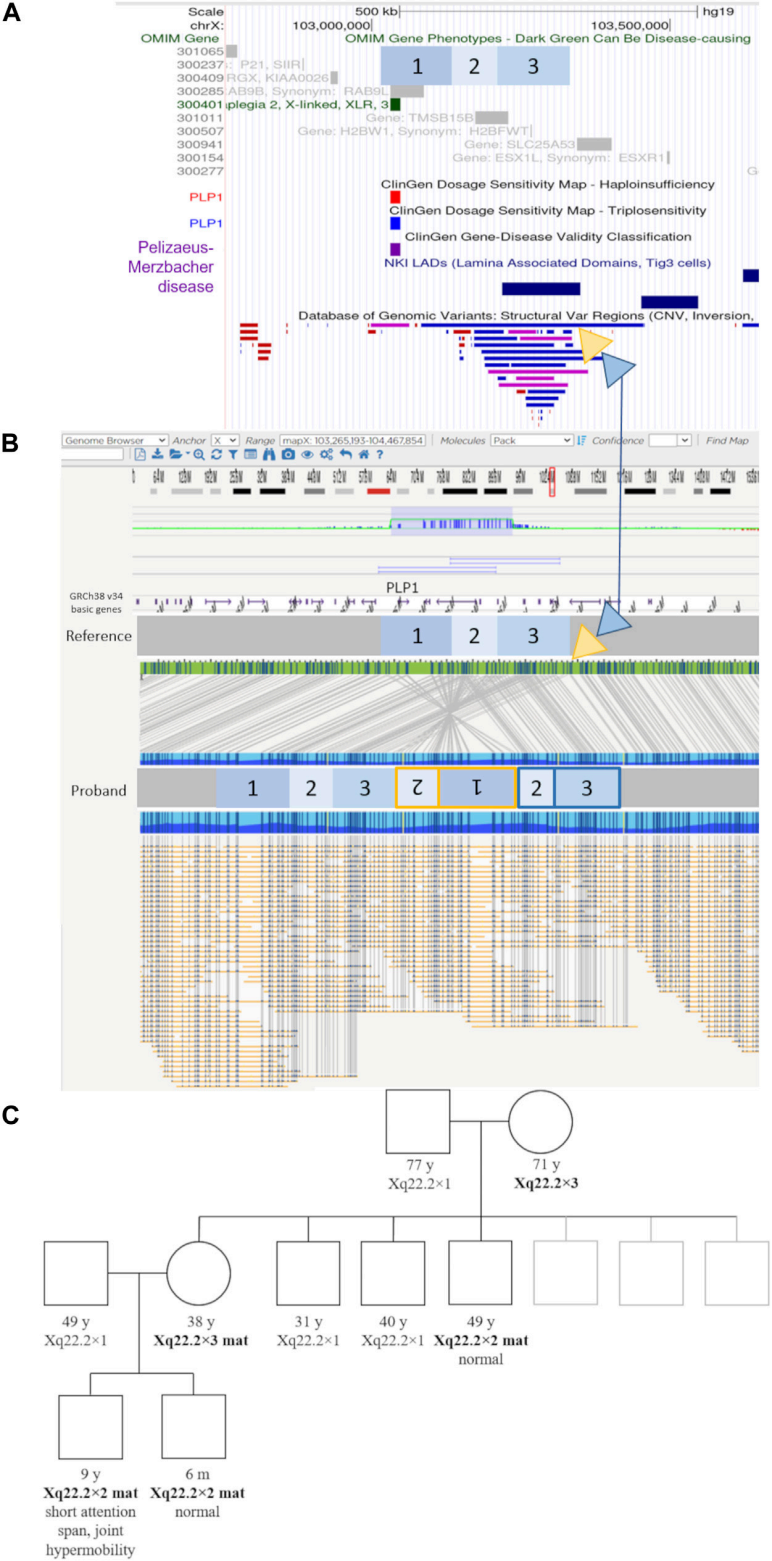
Genomic elements, optical genome mapping, and segregation results of the novel structural variant. **(A)** UCSC browser view showing the regions involved in the complex structural variants in blue boxes labeled 1 to 3, relative to genomic elements such as OMIM genes, LAD (lamina-associated domains), and DGV structural variants present in healthy individuals. Yellow and blue triangles show the location and genomic context of the inserted regions. The yellow triangle indicates the location of the inverted duplication (regions 1 and 2), while the blue triangle indicates the location of the tandem duplication regions (2 and 3), respectively (marked in the same color yellow/blue on the proband molecule map). **(B)** Optical genome mapping result and the corresponding schematic of the complex rearrangement in the proband. The involved regions are marked with numbers 1 to 3. Region 1 contains the *PLP1* gene. The yellow triangle indicates the location of the inverted duplication (regions 1 and 2), while the blue triangle indicates the location of the tandem duplication regions (2 and 3), respectively (marked in the same color yellow/blue on the proband molecule map). The duplicated region nr. three contains an additional lamina-associated domain. **(C)** The family pedigree based on the microarray testing results. Individuals with the familial structural variant are indicated in bold text.

The inverted duplication [GRCh38] Xq22.2(103734669_103958435)×2 contains the genes *PLP1* (OMIM:300401), *RAB9* (OMIM:300285), *TMSB15B* (OMIM:301011) and a lamina-associated domain (LAD), while the tandem duplication of the region [GRCh38] Xq22.2(103871512_104080423)×2, contains the gene *TMSB15B* (OMIM:301011) and same LAD domain, thus the inverted additional copy of the *PLP1* gene contains LAD domains at both ends (Figure 2, Supplementary Figure S2). The copy number gain of the *TMSB15B* was not detected by initial microarray of the proband due to lack of probes in the region.

The extended segregation analysis revealed one of the mother’s brothers, and the grandmother to be additional carriers of the inverted duplication (Figure 2, Supplementary Figure S1). None of the three adult carriers (mother, uncle, grandmother) had any signs or symptoms of neurological disease.

Based on this additional information, we estimated this *PLP1* inverted duplication variant is not associated with clinical observations in the proband and does not represent an increased risk for classic PMD. Accordingly, we have classified it as a variant of unknown significance (ACMG Criteria 1A, 2A, 4J, total points: 0.70) and deposited it in the ClinVar database (https://www.ncbi.nlm.nih.gov/clinvar/variation/1810757/). The mother continued with the pregnancy and gave birth at full term to a healthy boy. At 6 months old, her second son had a normal neurological examination, while the neurological status of the proband at his last examination at 9 years of age remains within normal, except for joint hypermobility, and he successfully attends regular school.

## Discussion

The pathogenicity of *PLP1* duplication has been well established in humans (Woodward et al., 1998; Mimault et al., 1999; Wolf et al., 2005; Regis et al., 2009) and *PLP1* overexpression as the underlying mechanism has been proposed based on animal models (Readhead et al., 1994; Peyron et al., 1997). All previously described *PLP1* duplications in males were reported to lead to central hypomyelination and progressive neurological deterioration with cognitive decline and spasticity, which are the hallmarks of PMD.

The discovery, by microarray, of a previously reported pathogenic duplication variant for a severe, progressive, early-onset disorder but an atypical clinical presentation in the proband, presented a unique prenatal counseling challenge.

Based on the atypical clinical presentation in the proband, we hypothesized that the microarray-identified duplication variant itself may not be typical. *PLP1*-related disorders of central nervous system myelin formation include a range of phenotypes from Pelizaeus-Merzbacher disease to spastic paraplegia 2 (Wolf et al., 1993). While all previous *PLP1* duplications in males were considered pathogenic, *PLP1* deletions in the context of various genomic rearrangements have already been reported to contribute to different dysmyelinating phenotypes (Inoue et al., 2002). So far, only one smaller, partially-overlapping inversion without an associated copy-number change, containing only the *PLP1* gene, has been identified in a presumably normal individual (Database of genomic variants, esv7229) (Ahn et al., 2009).

Because microarray analysis does not enable us to detect structural changes other than copy number variants, we examined the DNA of the patient using a novel technology, optical genome mapping. Optical genome mapping is based on the imaging of labeled ultra-high molecular weight DNA molecules and provides a high-resolution detection of structural variants such as inversions, insertions, and even balanced translocations. Indeed, optical genome mapping showed the *PLP1* variant in this family to be a previously undescribed complex structural variant—an inverted duplication, followed by a tandem duplication, located adjacent to the normal copy of the gene, and possessing putative lamina-associated domains at both ends (Figure 2, Supplementary Figure S2).

Although it remains unknown why this rare variant does not result in PMD, several genetic mechanisms are known to influence the expression of genes through topological means or rearrangement of regulatory regions, among which lamina-associated domains have been reported to actively inhibit transcription (Phillips-Cremins and Corces, 2013; van Steensel and Belmont, 2017; Huang et al., 2021; Hong and Cohen, 2022). While the mechanism by which the detected inverted duplication apparently avoids leading to the development of PMD currently remains unknown, the segregation analysis (Figure 2), showing three male carriers of this structural variant lacking clinical features associated with PMD, allows us to reclassify this variant from pathogenic to a variant of unknown significance on clinical criteria alone, with important implications in prenatal counseling and care. As the current molecular genetic testing of *PLP1* disorders consists of techniques not designed to detect orientation and not able to detect structural variants other than copy number variants, it is possible that similar rearrangements will be detected with increased use of novel technologies, such as optical genome mapping.

To conclude, we suggest that optical genome mapping may help both reclassify and reinterpret structural variants presenting as typical duplications on microarray, as it provides additional information regarding the orientation and genomic context of the copy-number change. Examining structural variants with such novel methods is warranted especially in cases with atypical clinical presentation and may in these cases lead to improved prenatal and postnatal genetic counseling.

## Materials and methods

### Patient data collection and consent

The clinical data were collected during the patients’ regular virtual counseling or in-person appointments, and all specialist examinations, including MRI, were performed as part of standard routine clinical care. Nine members of the family were tested using microarray, three of which were also tested using optical genome mapping, as part of routine genetic testing at the Clinical Institute of Genomic Medicine, University Medical Centre Ljubljana, Slovenia. Standard informed consent for this routine clinical genetic testing was obtained during their clinical examination or counseling visits, and written consent to participate in this study was provided by the participants’ legal guardiants/next of kin.

All procedures in the study were conducted according to the routine standard of care at the University Medical Centre Ljubljana, and in accordance with the principles of the Declaration of Helsinki.

### Microarray analyses

Microarray analysis was initially performed on the proband by using oligonucleotide array Agilent Technologies 4 × 180 K (AMADID:035689), according to the manufacturer’s instructions. Microarray analyses of other members of the family as well as the DNA sample isolated from amniotic fluid were performed by using the oligonucleotide array Agilent Technologies 8 × 60 K (AMADID: 031746) according to the manufacturer’s instructions. Agilent CytoGenomics 5.1.2.1 software was used to visualize and report the genomic gain in the Xq22.2.

The differences in the reported sizes of the duplicated region are due to the different resolutions of the chips used and were confirmed by additional microarray analyses (AMADID:035689, AMADID: 031746) to be concordant (Figure 1, Supplementary Figure S1).

### Optical genome mapping

High-weight molecular DNA was extracted from 1.5 million lymphocytes from whole blood (EDTA collected) using the SP Blood and Cell Culture DNA Isolation Kit following manufacturer instructions (Bionano, San Diego United States). The following day, DNA molecules were labeled using the DLS (Direct Label and Stain) DNA Labeling Kit (Bionan) with the DLE-1 enzyme. Labeled DNA was loaded on the three-flowcell Saphyr Chip^®^ (Part #20366) (Bionano) and ran on the Saphyr instrument (Bionano) to reach a minimum yield of 500 Gbp (DLE-1 label, [GRCh38] reference genome). The *de novo* assembly and Variant Annotation Pipeline were executed on Bionano Solve 3.7_03302022_283 while reporting and direct visualization of structural variants was done on Bionano Access 1.7.1.

The different sizes of this complex structural variant detected by both technologies are due to the differences between the resolution of the microarray probes and DLE-1 labeling sites of optical genome mapping.

### Variant interpretation

Variant interpretation of the microarray results was performed according to the ACMG in ClinGen guidelines (Riggs et al., 2020), by taking into account the following databases: GnomAD—(https://gnomad.broadinstitute.org/), Database of genomic variants (DGV)—(http://dgv.tcag.ca/gb2/gbrowse/dgv2_hg19/) (MacDonald et al., 2014) DECIPHER (https://www.deciphergenomics.org/), ClinVar (https://www.ncbi.nlm.nih.gov/clinvar/), and ClinGen (https://dosage.clinicalgenome.org/).

All figures were prepared from original visualizations generated by either the Agilent CytoGenomics5.1.1.15 software (Agilent technologies) or Bionano Access 1.7.2 software (Bionano), for microarrays and optical genome mapping, respectively. The UCSC Genome Browser Viewer was used to visualize the region of interest in the context of neighboring genomic regions (Nassar et al., 2023). The final composite Figures 1, 2 were technically prepared in terms of size, layout, format and type of file with no modification to original data, from the original visualizations, by using GIMP 2.10 (The GIMP Development Team, 2019).

## Data Availability

The datasets presented in this study can be found in online repositories. The names of the repository/repositories and accession number(s) can be found below: ClinVar database https://www.ncbi.nlm.nih.gov/clinvar/variation/1810757/.

## Supplementary material

SUPPLEMENTARY FIGURE S1 Results of microarray analysis in the extended family.

SUPPLEMENTARY FIGURE S2 Optical genome mapping results and comparison between mother, proband and their sibling.
