# Supplementary material for: Optical genome mapping in an atypical Pelizaeus-Merzbacher prenatal challenge
Source: Front Genet. 2023 Jul 25;14:1173426. doi: 10.3389/fgene.2023.1173426 (PMC10407396; doi:10.3389/fgene.2023.1173426)

OGM map of proband: location and size of complex inversion in Xq22.2

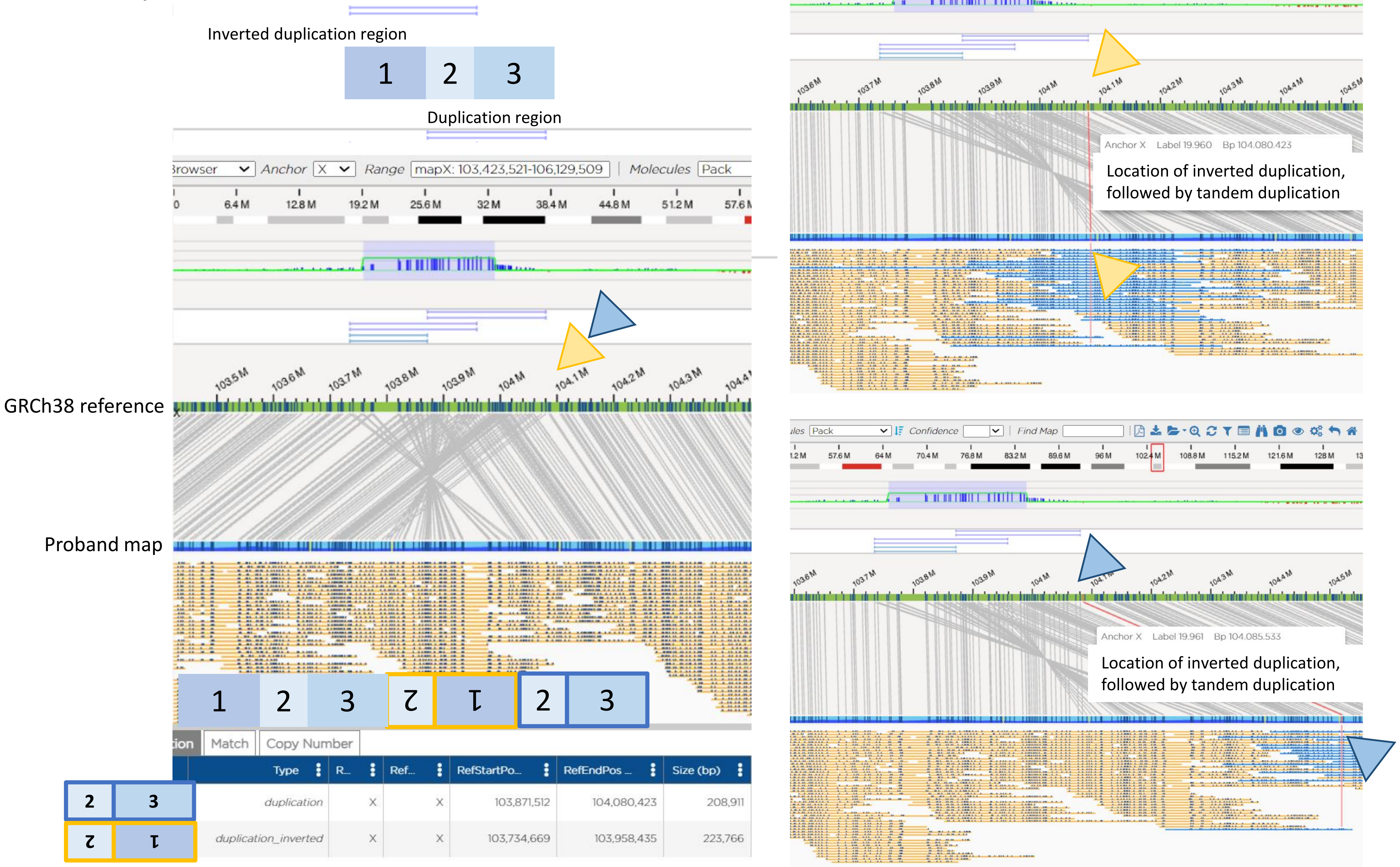

Type	R..	Ref..	RefStartPo..	RefEndPos ..	Size (bp)
duplication	X	X	103,871,512	104,080,423	208,911
duplication_inverted	X	X	103,734,669	103,958,435	223,766

OGM maps of proband and mother

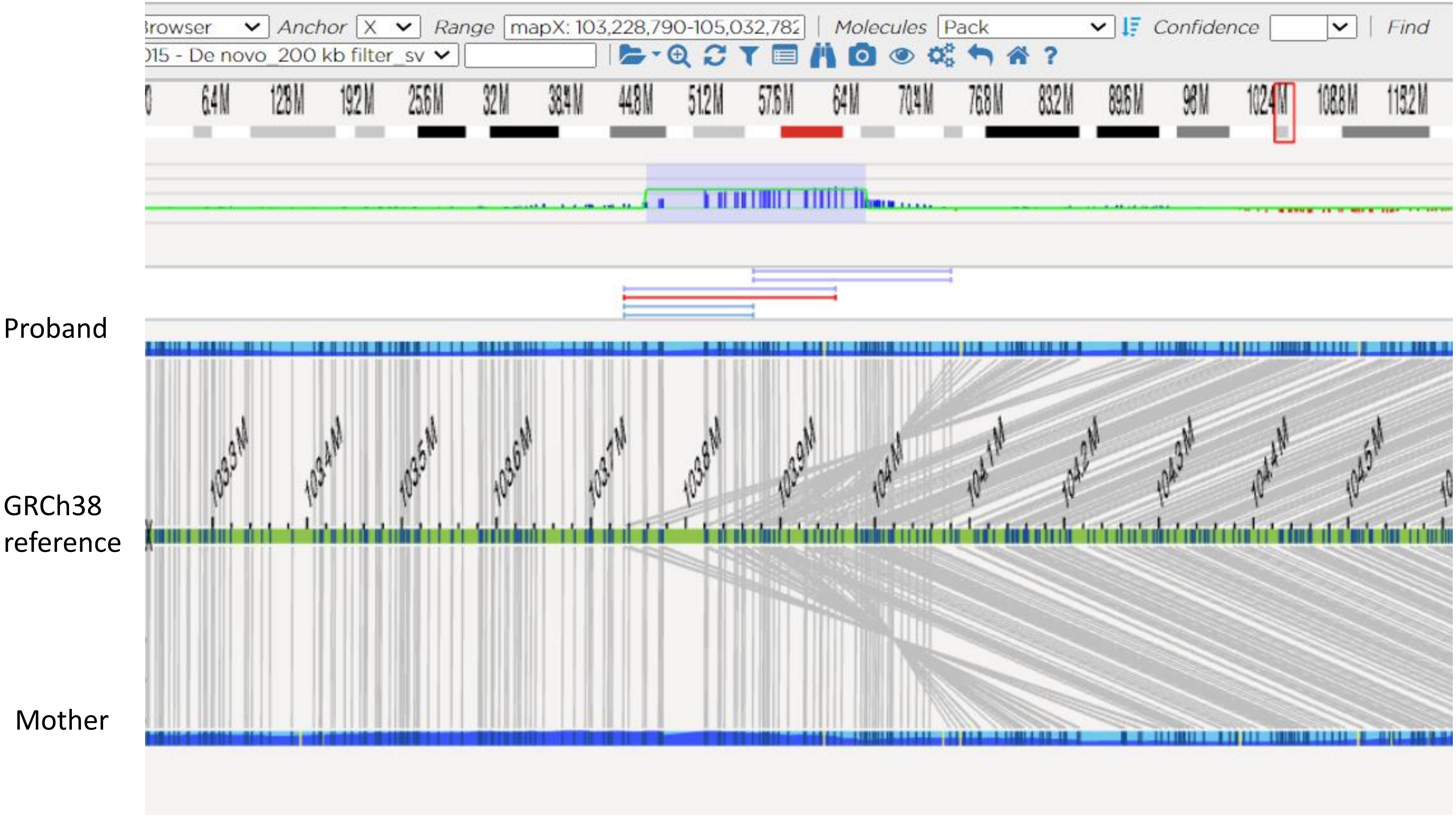

OGM maps of proband and younger brother (postnatal)

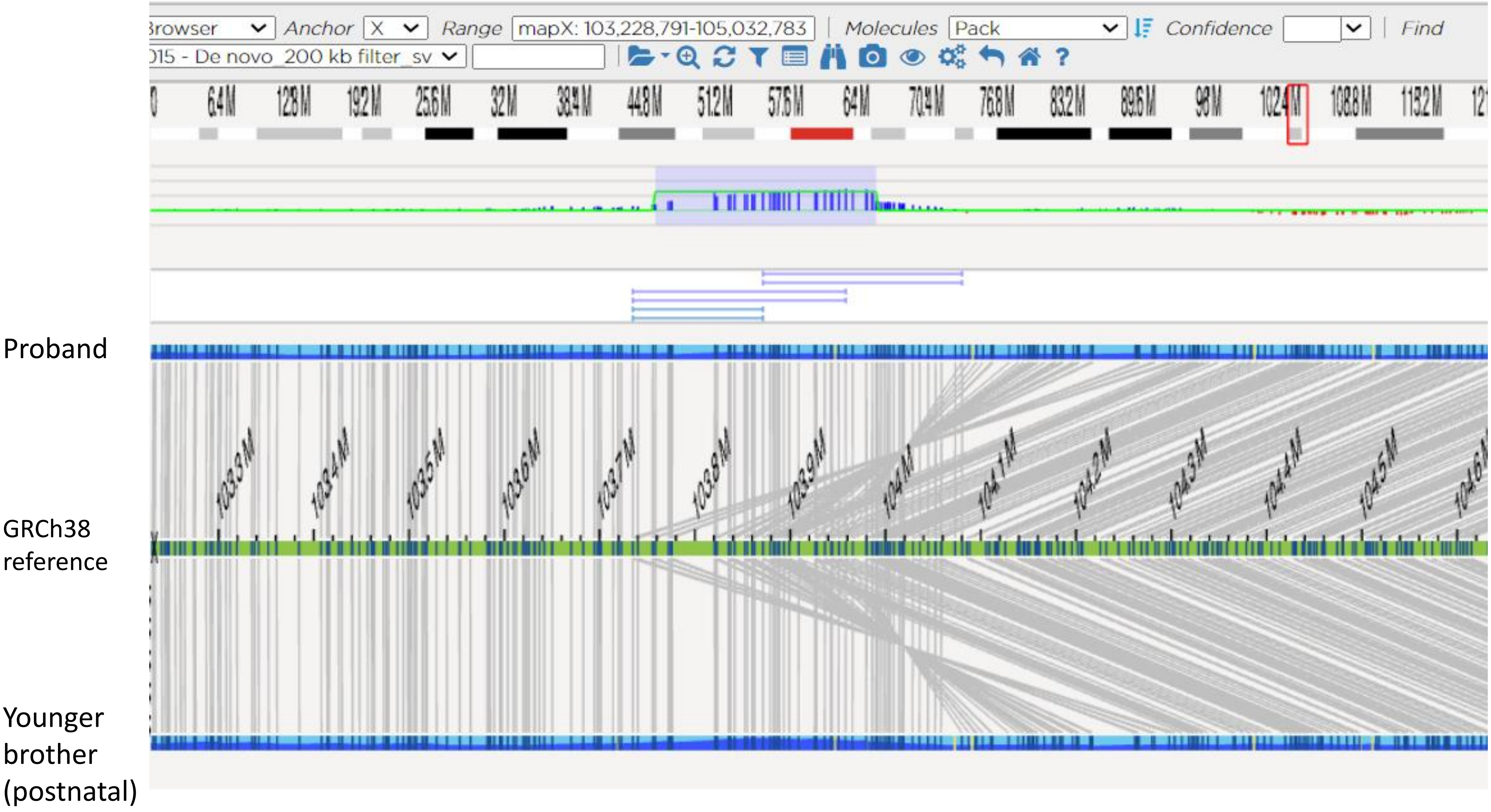

Supplement: Supplementary file 1 [file DataSheet2.pdf]
