# Supplementary material for: Optical genome mapping in an atypical Pelizaeus-Merzbacher prenatal challenge
Source: Front Genet. 2023 Jul 25;14:1173426. doi: 10.3389/fgene.2023.1173426 (PMC10407396; doi:10.3389/fgene.2023.1173426)
Supplement: Supplementary file 2 [file DataSheet1.pdf]

Proband (purple)<br>(AMADID:035689)	arr[GRCh37] Xq22.2(103008605_103172424)×2 mat<br>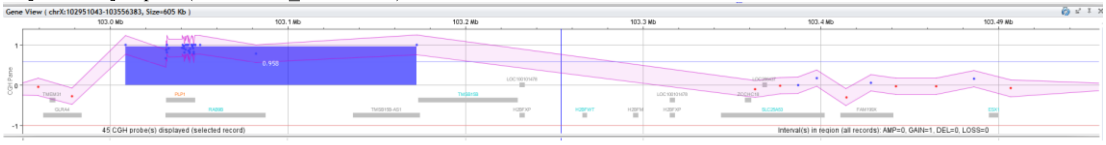
Proband (red)<br>(AMADID: 031746)<br>Control array<br><br>**Xq22.2×2 mat**	arr[GRCh37] Xq22.2(103003034_103288063)×2 mat<br>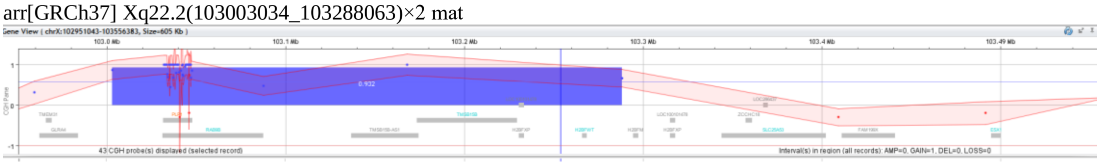
Prenatal:<br>(AMADID: 031746)<br><br>**Xq22.2×2 mat**	arr[GRCh37] Xq22.2(103003034_103288063)×2 mat<br>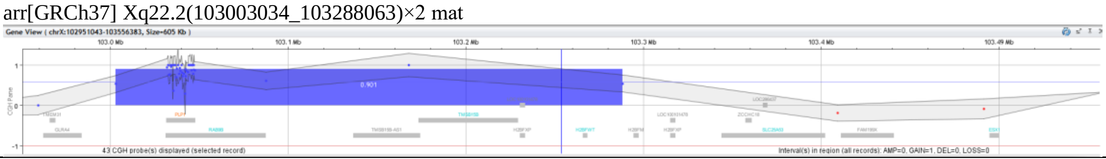
Uncle 3:<br>(AMADID: 031746)<br><br>**Xq22.2×2 mat**	arr[GRCh37] Xq22.2(103003034_103288063)×2 mat<br>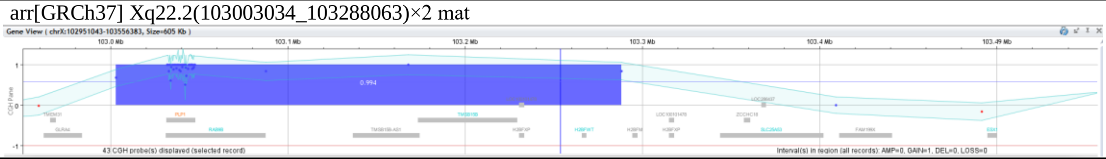
Mother :<br>(AMADID: 031746)<br><br>**Xq22.2×3 mat**	arr[GRCh37] Xq22.2(103003034_103167770)×3<br>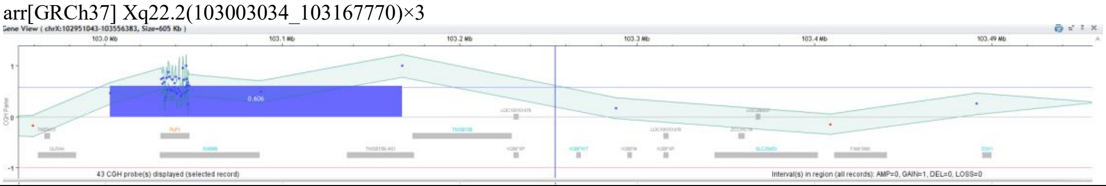
Grandmother<br>(AMADID: 031746)<br><br>**Xq22.2×3**	arr[GRCh37] Xq22.2(103003034_103288063)×3<br>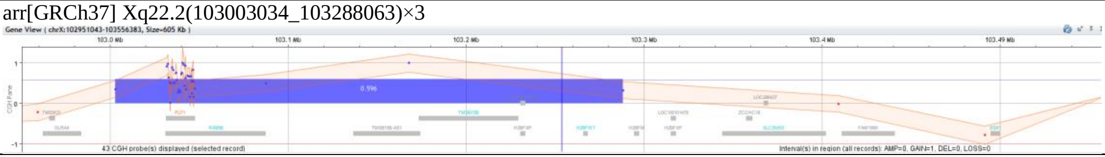
Father :<br><br>(AMADID: 031746)<br><br>Xq22.2×1	arr[GRCh37] Xq22.2(103003034_103288063)×1<br>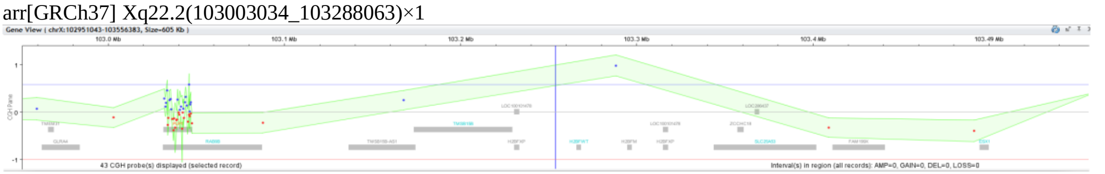
Grandfather:<br><br>(AMADID: 031746)<br><br>Xq22.2×1	arr[GRCh37] Xq22.2(103003034_103288063)×1<br>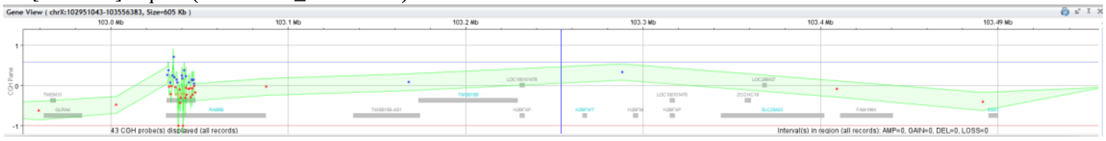
Uncle 1:<br>(AMADID: 031746)<br><br>Xq22.2×1	arr[GRCh37] Xq22.2(103003034_103288063)×1<br>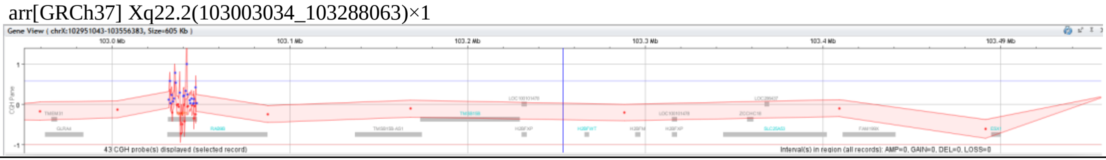
Uncle 2:<br>(AMADID: 031746)<br><br>Xq22.2×1	arr[GRCh37] Xq22.2(103003034_103288063)×1<br>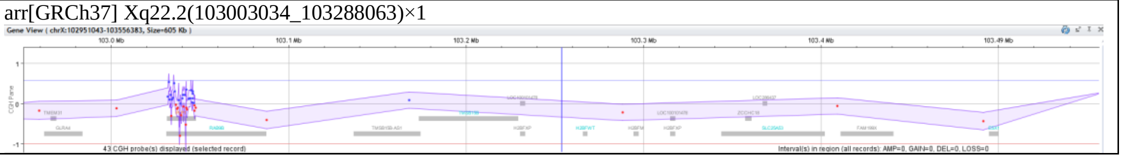
